# Meckel’s diverticulum causing intestinal obstruction in the newborn

**DOI:** 10.11604/pamj.2018.31.210.14840

**Published:** 2018-11-28

**Authors:** Mohamed Amine Oukhouya, Saad Andaloussi, Hicham Abdellaoui, Mohammed Tazi, Abdelhalim Mahmoudi, Aziz Elmadi, Khalid Khattala, Youssef Bouabdallah

**Affiliations:** 1 University Sidi Mohamed Ben Abdellah, CHU Hassan II, Fès, Morocco

**Keywords:** Intestinal obstruction, Meckel´s diverticulum, neonate

## Abstract

The causes of neonatal bowel obstruction are variable and dominated by malformations and the Meckel diverticulum must remain exceptional. We report a case of neonatal bowel obstruction in a six day old male neonate admitted on account of inability to pass stool, abdominal distension and bilious vomiting. The radiologic additional examinations are non-specific. Exploratory laparotomy found obstruction at the site of a Meckel's diverticulum.

## Introduction

Meckel's diverticulum (MD) is the most common congenital anomaly of the gastrointestinal tract, with a complication rate of approximately 4%. Bowel obstruction, gastrointestinal bleeding, acute inflammation and umbilical abnormalities are the most common presentations of MD in childhood, whereas symptomatic MD in the neonate is quite rare. We report an interesting case of symptomatic MD in newborn, only a similar case was found in the literature.

## Patient and observation

**Figure 1:**
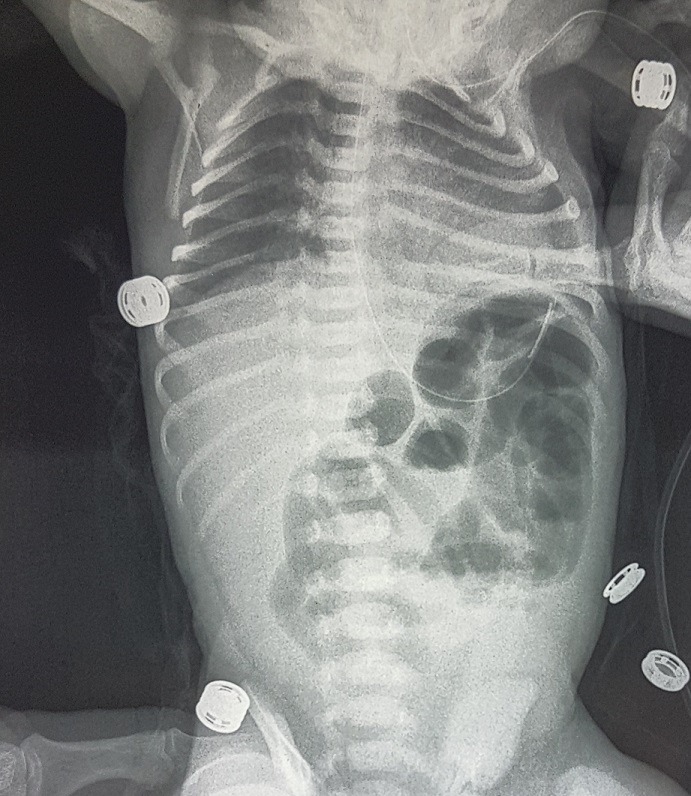
plain abdominal radiograph reveals air fluid levels and dilated loops of small bowel

**Figure 2:**
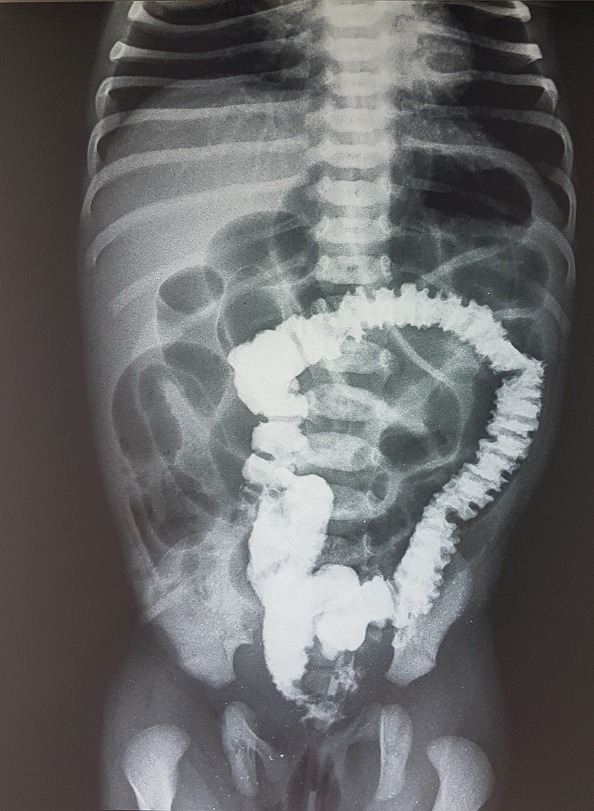
barium swallow shows a collapsed colon

**Figure 3:**
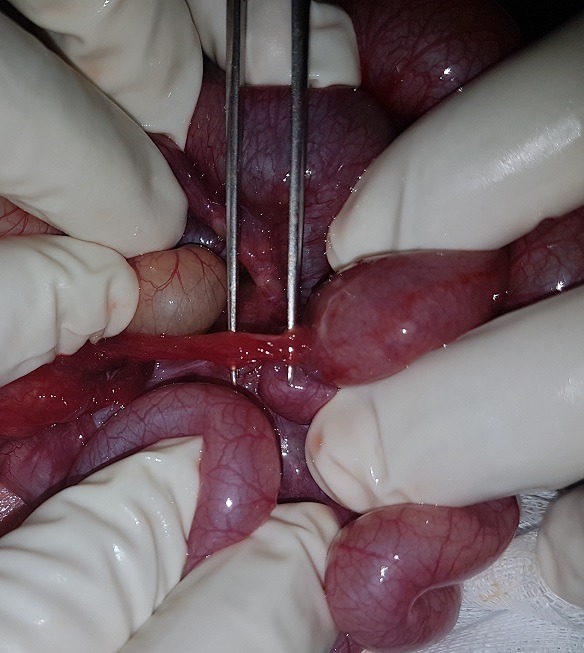
the tip of the diverticulum is attached to the mesentery

**Figure 4:**
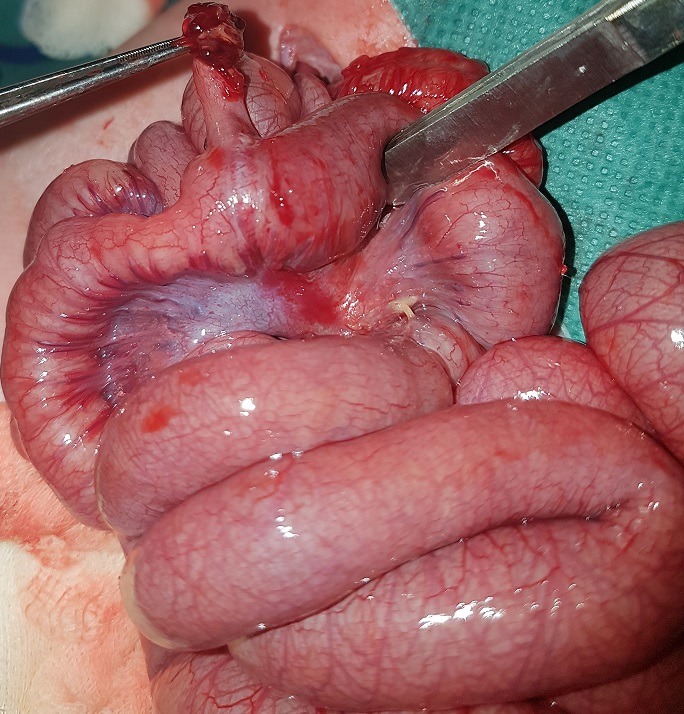
the site of a Meckel's diverticulum with marked dilatation of the bowel

A six day old male neonate, was admitted on account of inability to pass stool, abdominal distension and bilious vomiting. Symptoms had started 4 days previously. He had passed meconium within 24 h of birth and had been opening bowel regularly till the onset of symptoms. Physical examination revelated dehydrated and hypotonic term neonate with abdominal distension. Blood chemistry showed hyponatremia at 133 meq/l. Plain Abdominal radiograph revealed multiple air fluid levels and dilated loops of small bowel (Figure 1). An ultrasound was normal and a barium swallow showed a marked dilatation of the bowel and the colon was collapsed (Figure 2). Exploratory laparotomy found obstruction at the site of a Meckel's diverticulum with marked dilatation of the bowel. The diverticulum had encircled several loops of bowel and the tip was attached to the mesentery, thereby causing obstruction (Figure 3, Figure 4). Segmental resection of the MD and adjoining ileum was done and primary ileoileal anastomosis performed. Postoperative course was marked on the second day by the failure of the ileal anastomosis and an ileostomy was created, the post-operative course was good. The ileostomy closure will be done after a recovery period.

## Discussion

Although Meckel's diverticulum is the most common congenital malformation of the gastrointestinal tract, but neonatal revelation remains rare. Meckel's diverticulum originates from an incomplete obliteration of the omphalomesenteric or vitelline duct, which occurs around the fifth week of gestation [1, 2], it usually appears as a pouch, 3 to 6cm in length, arising from the antimesenteric border of the ileum at variable lengths from the ileocecal junction [1, 3]. The complications of Meckel's diverticulum in childhood are greatest in the first two years of life. Hemorrhage, secondary to peptic ulceration, is the most frequent manifestation. Next in frequency is intestinal obstruction, intussusception being the leading cause [4]. Intestinal obstruction accounts for 30 to 56% of symptomatic Meckel's diverticulum. Commonly documented mechanisms of obstruction are volvulus, intussusception, bands, Littre's hernia, internal hernias and strictures [5-8]. The plication mechanism reported in our case remains unusual. A diagnosis of neonatal bowel obstruction caused by Meckel's diverticulum is difficult to make [5, 8]. There are no distinguishing clinical features. Plain abdominal X-Ray is non-specific and will give features suggestive of small bowel obstruction [5, 8], ultrasonography may be more suggestive in cases where Meckel's diverticulum is complicated by volvulus, meconium impaction or intussusception [5, 6]. A laparotomy allows to identify the exact mechanism of the intestinal obstruction caused by the Meckel diverticulum, and to remove the obstacle. Mortality statistics on the surgically treated complications of Meckel's diverticulum in childhood average between 15% and 20%.

## Conclusion

Meckel's diverticulum is a frequent malformation but neonatal revelation remains exceptional. No investigation can be made to establish the diagnosis, but only a laparotomy can identify the lesion and the exact mechanism to propose an adequate treatment.

## Competing interests

The authors declare no conflict of interest.
